# Disaster Preparedness among Emergency Medical Service Providers: A Systematic Review Protocol

**DOI:** 10.1155/2020/6102940

**Published:** 2020-10-26

**Authors:** Mehdi Beyramijam, Hamid Reza Khankeh, Mehrdad Farrokhi, Abbas Ebadi, Gholamreza Masoumi, Mohsen Aminizadeh

**Affiliations:** ^1^Health in Emergency and Disaster Research Center, The University of Social Welfare and Rehabilitation Sciences, Tehran, Iran; ^2^Department of Clinical Science and Education, Karolinska Institute, Stockholm, Sweden; ^3^Behavioral Sciences Research Center, Life Style Institute, Baqiyatallah University of Medical Sciences, Tehran, Iran; ^4^Nursing Faculty, Baqiyatallah University of Medical Sciences, Tehran, Iran; ^5^Emergency Management Research Center, Iran University of Medical Sciences, Tehran, Iran

## Abstract

*Background*. The emergency medical service (EMS) provides first-line medical care to people who require urgent medical care in emergency and disaster situations. Preparedness is the most effective approach for the management of disaster risks, and it is essential for the emergency medical service (EMS) providers, such as paramedics, emergency medical technicians (EMT), and other EMS personnel. This systematic review will explore evidence on the preparedness of emergency medical service providers in emergency and disaster situations by reviewing peer-reviewed journal articles. *Methods/Design*. This study will be conducted on peer-reviewed articles published between 2005 and 2019 to explore the preparedness of emergency medical service providers in emergencies and disasters. Scopus, Web of Science, PubMed, and Google Scholar will be thoroughly searched to identify published studies on emergency and disaster preparedness. The following keywords will be used for searching the databases: “Medical Technician,” “Paramedic,” “Emergency Paramedic,” “Emergency Medicine Technician,” “Emergency Medical Technician,” “Emergency Prehospital Provider,” “Emergency Preparedness,” “Disaster Preparedness,” “Hospital Preparedness,” “Disaster management,” “Disaster Competencies,” “Disaster Readiness,” “Disaster,” “Disaster Role,” “Readiness, Preparedness, Terrorist,” “Mass Casualty Incident,” “Major incidents,” “Mass Casualty,” “Mass Gathering,” “CBRNE,” “Weapons of Mass Destruction,” and “Chemical, Biological, Radiological, Nuclear, and Explosive Event.” *Discussion*. To the best of our knowledge, no comprehensive review study has been conducted on the preparedness of emergency medical service providers in disaster situations. This study is the first attempt to address this gap. It will also explore the key dimensions in disaster preparedness of EMS providers and the strategies to enhance their preparedness. Identifying the key dimensions of disaster preparedness is the first step in designing and developing valid instruments to evaluate EMS provider's disaster preparedness and as well as adopting appropriate strategies to improve the level of their preparedness (This systematic review is registred in PROSPERO with CRD42020149689).

## 1. Background

The emergency medical service (EMS) provides first-line medical care to victims who need emergent and urgent medical care during emergency and disaster situations. The WHO considers EMS as an integral part of any effective and efficient health system [1]. Disasters, especially natural disasters, are inevitable and occur almost everywhere in the world and affect communities [2]. In 2018, 281 climate-related and geophysical events occurred in the world [3], and at the time of writing this paper (22 Aug 2020), the Covid-19 pandemic has caused the death of 848,484 people in the world [4]. As a simple definition, any event that overwhelms existing societal systems is a disaster [1]. The United Nations Office for Disaster Risk Reduction (UNISDR) defines a disaster as a serious disruption of the functioning of a community or a society due to hazardous events interacting with conditions of vulnerability and exposure, leading to widespread human, material, economic and environmental losses, and impacts [5]. Given the widespread occurrence of emergencies and disasters, managing their impacts, particularly health impacts, is essential, and it has been advocated by international organizations such as the Sendai Framework for Disaster Risk Reduction (SFDRR) 2015–2030 [6]. Preparedness is one of the main phases and components of disaster risk management [7], and the SFDRR 2015–2030 emphasizes an increase in preparedness as part of the first five main priorities (priority # 4) of disaster risk reduction strategy [6]. Preparedness is considered as an outcome and a goal of disaster risk reduction (DRR). The health systems as one of the main pillars of the communities play the most important role in disaster risk reduction. Hence, special consideration of the preparedness of the health systems, especially EMS systems is essential to ensure the implementation of the SFDRR 2015–2030 [8]. The EMS systems provide first-line medical care for emergency and disaster victims worldwide [1]. Historically, the civilian experiences, and especially the experiences of caring for the casualties of past wars, have played a significant role in shaping current EMS principles and methods [1]. The US Civil War, for example, was largely the starting point for EMS systems in the United States [9]. Over the last 50 years, the emergency medical service systems have developed around the world, and they are developing today [1]. With the spread of major emergencies and disasters around the world, the need for prehospital emergency care services has been further strengthened and concluded that EMS providers need to be more trained and prepared for a coordinated and efficient response [1]. EMS providers are the first healthcare providers to be present in the natural disaster fields, and they are also present at manmade disasters fields such as chemical, biological, radiological, and nuclear sites and explosive-related agents (CBRNE) which are potential targets of terrorist attacks [10]. They play an important role in the planning, response, and recovery from emergency and disasters, and according to the United State Department of Homeland Security (USDHS), the incident management, triage, prehospital treatment, management and distribution of medical equipment, prevention, care, and protection of the injured people are among the duties of the EMS in various emergencies and disaster situations [11]. Investigating the current state of disaster preparedness and the capabilities of the EMS providers in response to emergency and disasters could be a major step towards enhancing the outcome and recovery from disasters. Lack of preparedness of the EMS providers leads to the undesirable outcome and may impede effective recovery from disasters in the communities [12]. Therefore, this systematic review will be conducted to explore the levels and key dimensions of the emergency medical service providers' preparedness in response to major emergencies and disasters to provide insights into their preparedness in handling disaster situations.

## 2. Methods/Design

### 2.1. Research Questions

The primary objectives of this systematic review include the following:

1. What are the preparedness level of EMS providers (i.e., EMTs, paramedics, nurses, physicians, and other EMS professional personnel that work in prehospital setting) in response to major emergencies and disasters?
2. What are the key dimensions of EMS providers' preparedness for response to major emergencies and disasters?
3. What are the strategies to enhance EMS providers' preparedness for response to major emergencies and disasters?

### 2.2. Systematic Review

This systematic review study will be conducted using the PRISMA protocol (PRISMA-P 2015) [13]. This protocol is developed using the PRISMA protocol checklist and has been submitted at PROSPERO with the submission number 149689.

## 3. Eligibility Criteria

### 3.1. Inclusion and Exclusion Criteria

In this study, English studies including initial studies (i.e., qualitative, observational, and interventional studies) and secondary studies (i.e., systematic review, narrative review, and meta-analysis) published between 2005 and 2020 and their objectives to measure disaster preparedness of emergency medical service providers will be included. Also, the high-quality postincident reviews and the actions report about major incidents and disasters that are in the grey literature (i.e., the conference papers, theses and dissertations, the websites of identified authorities, and other grey literature sources) and published following independent investigations will be included. The studies that (1) did not report findings on disaster preparedness of EMS providers, (2) included EMS providers as part of a sample with other professionals, (3) reported findings on EMS providers' preparedness in situations other than emergencies and disasters and, (4) which are not published or do not have the abstract and full text, and (5) book chapters, dissertations/theses, and conference papers will be excluded.

### 3.2. Type of Participants

The study participants include all the emergency medical personnel such as emergency medical technicians (EMTs), paramedics, ambulance technicians, EMS nurses, and EMS physicians. The study imposes no age, gender, ethnicity, and other population characteristic restrictions.

### 3.3. Information Sources and the Search Strategy

The electronic databases such as PubMed, Web of Science Core Collection, Scopus, and Google Scholar will be searched to access the relevant studies. For searching each database, initially, the keywords are determined, and their synonyms are specified using MESH. Then, English keywords and their combination will be searched in the aforesaid databases based on title tag, abstract, and keywords from 2005 to 2019. The syntax searched in the databases to obtain relevant studies will be as follows.

### 3.4. PubMed

The search terms used are as follows: “Medical Technician^*∗*^”[tiab] OR Paramedic^*∗*^[tiab] OR “Emergency Paramedic^*∗*^”[tiab] OR “Emergency Medicine Technician^*∗*^”[tiab] OR “Emergency Medical Technician^*∗*^”[tiab] OR “Emergency Prehospital Provider^*∗*^”[tiab]) AND (“Emergency Preparedness”[tiab] OR “Disaster Preparedness”[tiab] OR “Hospital preparedness”[tiab] OR “Disaster management”[tiab] OR “disaster competencies”[tiab] OR “disaster readiness”[tiab] OR Disaster^*∗*^[tiab] OR “disaster role”[tiab] OR readiness[tiab] OR preparedness[tiab] OR CBRNE[tiab] OR Terrorist^*∗*^ OR “Mass casualty incident^*∗*^”[tiab] OR “Major incident^*∗*^”[tiab] OR “Mass Casualty” OR “mass gathering^*∗*^”[tiab] OR “Weapons of Mass Destruction”[tiab] OR Chemical[tiab] OR Biological[tiab] OR Radiological[tiab] OR Nuclear[tiab] OR Explosive[tiab].

### 3.5. Scopus

The search terms used are as follows: TITLE-ABS-KEY (“Medical Technician^*∗*^”) OR TITLE-ABS-KEY (Paramedic^*∗*^) OR TITLE-ABS-KEY (“Emergency Paramedic^*∗*^”) OR TITLE-ABS-KEY (“Emergency Medicine Technician^*∗*^”) OR TITLE-ABS-KEY (“Emergency Medical Technician^*∗*^”) OR TITLE-ABS-KEY (“Emergency Prehospital Provider^*∗*^”) AND TITLE-ABS-KEY (“Emergency Preparedness”) OR TITLE-ABS-KEY (“Disaster Preparedness”) OR TITLE-ABS-KEY (“Hospital preparedness”) OR TITLE-ABS-KEY (“Disaster management”) OR TITLE-ABS-KEY (“disaster competencies”) OR TITLE-ABS-KEY (“disaster readiness”) OR TITLE-ABS-KEY (Disaster^*∗*^) OR TITLE-ABS-KEY (“disaster role”) OR TITLE-ABS-KEY (readiness) OR TITLE-ABS-KEY (preparedness) OR TITLE-ABS-KEY (CBRNE) OR TITLE-ABS-KEY (Terrorist^*∗*^) OR TITLE-ABS-KEY (“Mass casualty incident^*∗*^”) OR TITLE-ABS-KEY (“Mass Casualty”) OR TITLE-ABS-KEY (“Major incident^*∗*^”) OR TITLE-ABS-KEY (“mass gatherings^*∗*^”) OR TITLE-ABS-KEY (“Weapons of Mass Destruction”) OR TITLE-ABS-KEY (Chemical) OR TITLE-ABS-KEY (Biological) OR TITLE-ABS-KEY (Radiological) OR TITLE-ABS-KEY (Nuclear) OR TITLE-ABS-KEY (Explosive).

### 3.6. Web of Science

The search terms used are as follows: TS = (“Medical Technician^*∗*^” OR Paramedic^*∗*^ OR “Emergency Paramedic^*∗*^” OR “Emergency Medicine Technician^*∗*^” OR “Emergency Medical Technician^*∗*^” OR “Emergency Prehospital Provider^*∗*^”) AND TS = (“Emergency Preparedness” OR “Disaster Preparedness” OR “Hospital preparedness” OR “Disaster management” OR “disaster competencies” OR “disaster readiness” OR Disaster^*∗*^ OR “disaster role” OR readiness OR preparedness OR CBRNE OR Terrorist^*∗*^ OR “Mass casualty incident^*∗*^” OR “Major incident^*∗*^” OR “Mass Casualty” OR “mass gathering^*∗*^”OR “Weapons of Mass Destruction” OR Chemical OR Biological OR Radiological OR Nuclear OR Explosive.

### 3.7. Methods for Study Selection

After searching all the databases, the selected articles will be entered into EndNote, and duplicates will be removed. In the next step, the titles and abstracts will be reviewed to find relevant studies. Following and determining the relevant studies, two well-informed reviewers specialized in the field independently study the full text of the articles. Any potential disagreement between the two reviewers will be resolved through a group discussion and consensus. Also, in the case of unresolved disagreement, a third reviewer will be invited for assistance. Furthermore, to find other potentially relevant articles, the references of the extracted articles will be investigated. Using the remarks made by specialists in this field, the credible journals related to this field will also be manually reviewed to find relevant studies published between 2005 and 2019 (see additional file 2: flow chart of the selection process (available here)).

### 3.8. Methods for Data Extraction

Data will be extracted by a full-text review of the selected articles. The extracted data will include specific details about (1) country, (2) research methodology, (3) research instruments, (4) reported level of disaster preparedness of EMS providers, (5) the main component and dimensions of EMS providers' disaster preparedness, and (6) the strategies to enhance EMS providers' preparedness for response to major emergency and disasters. Also, through the reviewer's consensus, other details will be extracted from the selected article.

### 3.9. Quality Assessment

Due to the inclusion of studies with different methodologies, no unique tools for quality assessment can be presented in this step. Therefore, to evaluate the methodological quality of the included studies, nevertheless, quality assessment tools introduced in the Strengthening the Reporting of Observational Studies in Epidemiology (STROBE) will be used depending on the type of the study. In this step, the articles will be assessed by two independent reviewers following its proper assessment tool for determining the eligibility of the articles for inclusion. Any disagreements will be resolved through a group discussion or by a third reviewer.

### 3.10. Data Analysis and Data Synthesis

Given our current knowledge of the literature, diversity of the samples, and variances in the data collection method, the use of statistical methods such as quantitative meta-analysis for the analysis of the data would not be possible. Hence, the thematic analysis technique (Center for Reviews and Dissemination, 2008) will be used for the analysis of the extracted data.

## 4. Discussion

Preparedness is one of the most important strategies for disaster risk management based on international documents such as SFDRR 2015–2030 [6]. Hence, the preparedness of healthcare professionals, especially EMS providers, is critical. A review of the literature shows that extensive studies have been conducted on the preparedness status of other healthcare workers in disaster situations [2, 14–16]. However, to date, there is no comprehensive study conducted on the disaster preparedness status of EMS providers. This systematic review will address this gap by bringing together evidence in this area. It will also explore the key dimensions in disaster preparedness of EMS providers and the strategies to enhance their preparedness. Identifying the key dimensions of disaster preparedness is the first step in designing and developing valid instruments to evaluate EMS provider's disaster preparedness and as well as adopting appropriate strategies to improve the level of their preparedness. Therefore, the results of this study will provide valuable information for EMS officers, administrators, and researchers in an attempt to enhance EMS provider's preparedness and the outcome of the EMS system in emergency and disaster situations.

## Data Availability

The data used to support the findings of this study are available from the corresponding author upon request.

## Abbreviations

EMS: Emergency medical services
EMT: Emergency medical technicians
CBRNE: Chemical, biological, radiological, nuclear, and explosive
MCI: Mass casualty incident
WHO: World Health Organization
UNDRR: United Nations Office for Disaster Risk Reduction
US: United States
PRISMA: Preferred reporting items for systematic reviews and meta-analyses
MESH: Medical subject headings.

## Supplementary Materials

Supplementary Materials Supplementary 1. File 1: selection process flow chart. Supplementary 2. File 2: PRISMA-P + checklist.

## References

[1] Ciottone G. R., Biddinger P. D., Darling R. G. (2015). *Ciottone’s Disaster Medicine*.

[2] Labrague L. J., Hammad K., Gloe D. S. (2018). Disaster preparedness among nurses: a systematic review of literature. *International Nursing Review*.

[3] Sapir G. (2018). *Extreme Weather Events Affected 60 Million People*.

[4] Coronavirus Cases, 2020, https://www.worldometers.info/coronavirus/

[5] UNISDR (2015). *Proposed Updated Terminology on Disaster Risk Reduction—A Technical Review 2015*.

[6] UNO (2015). *Framework for Disaster Risk Reduction 2015–2030*.

[7] Asghar S., Alahakoon D., Churilov L. (2006). A comprehensive conceptual model for disaster management. *Journal of Humanitarian Assistance*.

[8] Lo S. T. T., Chan E. Y. Y., Chan G. K. W. (2017). Health emergency and disaster risk management (health-EDRM): developing the research field within the Sendai framework paradigm. *International Journal of Disaster Risk Science*.

[9] Barton C. (1907). *The Story of My Childhood*.

[10] Stevens G., Jones A., Smith G. (2010). Determinants of paramedic response readiness for CBRNE threats. *Biosecurity and Bioterrorism: Biodefense Strategy, Practice, and Science*.

[11] Udoh S. (2007). *Target Capabilities List: A Companion to the National Preparedness Guidelines*.

[12] Elliott R. W. (2010). *Measuring Disaster Preparedness of Local Emergency Medical Services Agencies*.

[13] Moher D., Shamseer L., Clarke M. (2015). Preferred reporting items for systematic review and meta-analysis protocols (PRISMA-P) 2015 statement. *Systematic Reviews*.

[14] Johnstone M.-J., Turale S. (2014). Nurses’ experiences of ethical preparedness for public health emergencies and healthcare disasters: a systematic review of qualitative evidence. *Nursing & Health Sciences*.

[15] Chaffee M. (2009). Willingness of health care personnel to work in a disaster: an integrative review of the literature. *Disaster Medicine and Public Health Preparedness*.

[16] Hansoti B., Kellogg D. S., Aberle S. J. (2016). Preparing emergency physicians for acute disaster response: a review of current training opportunities in the US. *Prehospital and Disaster Medicine*.

