# Supplementary material for: Disaster Preparedness among Emergency Medical Service Providers: A Systematic Review Protocol
Source: Emerg Med Int. 2020 Oct 26;2020:6102940. doi: 10.1155/2020/6102940 (PMC7683168; doi:10.1155/2020/6102940)
Supplement: Supplementary Materials — Supplementary 1. File 1: selection process flow chart. Supplementary 2. File 2: PRISMA-P + checklist. [file 6102940.f1.zip › 6102940.f1/Suplementary file 1.docx]

Supplementary material 1: Selection process flow chart

Titles will be excluded (n =?)

## Included

Studies will be included in quantitative synthesis
(n =? )

## Eligibility

Full-text articles will be assessed for eligibility
(n = ?)

Full-text articles will be excluded, with reasons

(n =?)

## Screening

Records will be identified through database searching
(PubMed; Scopus; Web of Science; Google scholar)

n =? ))

## Identification

Duplicate will be removed
(n = ? )

Records after duplicates removed
(n =?)

Records will be screened
(n =? )

Records will be excluded

(abstract analysis)
(n = ? )
